# Mesenchymal Stem Cells as New Therapeutic Approach for Diabetes and Pancreatic Disorders

**DOI:** 10.3390/ijms19092783

**Published:** 2018-09-16

**Authors:** Arianna Scuteri, Marianna Monfrini

**Affiliations:** Experimental Neurology Unit and Milan Center for Neuroscience, School of Medicine and Surgery, University Milano-Bicocca, Via Cadore 48, 20900 Monza, Italy; marianna.monfrini@unimib.it

**Keywords:** diabetes, acute pancreatitis, chronic pancreatitis, pancreatic cancer, mesenchymal stem cells

## Abstract

Diabetes is a worldwide disease which actually includes different disorders related to glucose metabolism. According to different epidemiological studies, patients affected by diabetes present a higher risk to develop both acute and chronic pancreatitis, clinical situations which, in turn, increase the risk to develop pancreatic cancer. Current therapies are able to adjust insulin levels according to blood glucose peak, but they only partly reach the goal to abrogate the consequent inflammatory milieu responsible for diabetes-related diseases. In recent years, many studies have investigated the possible use of adult mesenchymal stem cells (MSCs) as alternative therapeutic treatment for diabetes, with promising results due to the manifold properties of these cells. In this review we will critically analyze the many different uses of MSCs for both diabetes treatment and for the reduction of diabetes-related disease development, focusing on their putative molecular mechanisms.

## 1. Introduction

Pancreatic disorders are a group of diseases which may affect the exocrine as well as the endocrine portion of the organ, with different severity degrees and, overall, different prognosis. They include diabetes, acute and chronic pancreatitis, and pancreatic cancer, distinct diseases which can, however, become strictly correlated, since diabetes can rise as a consequence of acute and chronic pancreatitis, and diabetic patients present an increased risk to develop acute pancreatitis [1]. Both of these conditions lead, in turn, to a higher risk to develop pancreatic cancer, in particular pancreatic ductal adenocarcinoma, the most common form [2]. Due to the heavy consequences of all such diseases affecting the pancreas and, overall, to the lack of effective therapeutic options, especially for pancreatitis and pancreatic cancer, the researchers have increased their efforts to provide a valid alternative therapy, by focusing on regenerative medicine and, in particular, on the use of mesenchymal stem cells (MSCs). Concerning diabetes, in the last decade the hypothesis to use MSCs gained ground due to their features (described later), which could theoretically be useful against the etiopathological mechanisms of such disease. Recently, it has been suggested that MSCs could tackle the issue from more than one front, and the use of MSCs has also been suggested to treat pancreatitis and pancreatic cancer, with different aims but, in any cases, with promising results.

In the following sections, after an overview on pancreatic disorders and their linking connections, we will present the most significant studies on the potential use of MSCs to fight pancreatic diseases, with a critical examination of the supporting data and of their mechanisms of action.

## 2. Pancreatic Disorders

### 2.1. Diabetes

Diabetes refers to different glucose metabolism disorders that, according to the American Diabetes Association (ADA), can be classified into: type-I, characterized by insulin deficiency, due to autoimmune β-cell destruction; type-II, characterized by insulin resistance that lead to abnormal insulin secretion; other forms (often reported as type-III), due to different causes later specified, and gestational diabetes (also known as type-IV), which is a temporary disorder and the most frequent health problem during pregnancy. Type-III diabetes includes many forms with different causes: IIIa, caused by genetic defects of β-cell function; IIIb, due to genetic defects in insulin action; IIIc, for the diseases of exocrine pancreas; IIId for endocrinopathies; IIIe, drug- or chemical-induced; IIIf, an infection consequence; IIIg, an uncommon form of immune-mediated diabetes; and IIIh, other genetic syndromes sometimes associated with diabetes [3], as reported in Table 1. All these different diabetes forms have very different etiology; nevertheless, they share a common symptom: hyperglycemia.

**Figure 1:**
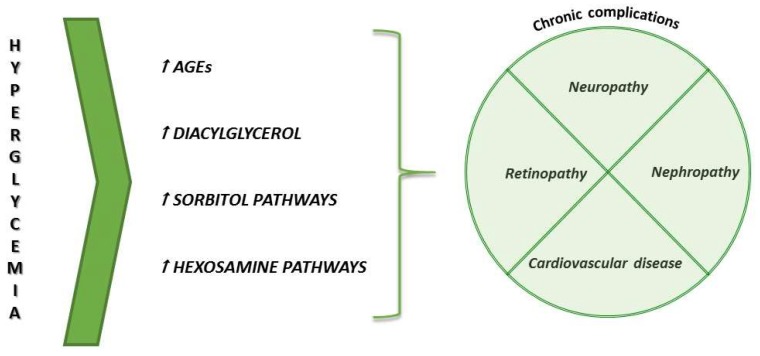
Summary of the theories underlying long-term complications of diabetes.

Hyperglycemia is the trigger factor that leads to organ dysfunction and long-term complications, such as retinopathy, nephropathy, neuropathy, and cardiovascular disease, and different theories have been proposed to explain their onset, as reported in Figure 1. The first one is based on the formation of advanced glycosylation end products (AGEs), which can cause many dysfunctions in both intra- and extracellular compartments. In particular AGEs are able to cross-link polypeptides of the same protein, such as collagen, to confer resistance to proteolytic digestion, and to link nucleic acids, thus inducing pathogenic modifications [4]. Another theory takes into account sorbitol increase: due to an excess of glucose, a part of it is metabolized by aldose reductase into sorbitol. Such modified sorbitol concentration increases intracellular osmolarity and alters redox potential, with ROS generation leading to general cellular dysfunction [4]. It has been also suggested that hyperglycemia leads to diacylglycerol increase, which actives protein kinase C (PKC) that, in turn, alters collagen and extracellular matrix proteins. Furthermore, PKC activates NADPH-oxidase, with consequent ROS production, cellular oxidative stress and, in the end, cell death [5]. The last hypothesis suggests that the activation of hexosamine pathway leads to fructose-6-posphate generation, a substrate for O-linked glycosylation. Such a modification can be responsible for protein inhibition, such as it occurs for key insulin signaling proteins Akt, pancreatic duodenal homeobox-1 (PDK1), and for glucose transporter 4 (GLUT-4) [6].

The control of blood glucose level into a physiological range is the goal of the therapy for all diabetes forms, and glycemia compensation is mandatory for the prevention or the delay of long term complications onset.

Next to well-known complications, diabetic patients present a higher risk to develop both acute and chronic pancreatitis, clinical situations which, in turn, increase the risk to develop pancreatic cancer. A bidirectional correlation has been established between types-I, -II, and -III diabetes and the other pancreatic disorders, i.e., according to the diabetes form, pancreatitis could come as a consequence or it could be the direct cause.

### 2.2. Pancreatitis

#### 2.2.1. Acute Pancreatitis

Acute pancreatitis is an inflammatory condition of acinar cells due to a wrong or a premature activation of pancreatic enzymes inside the pancreas instead of intestinal lumen. In order to avoid autodigestion of the pancreas, in healthy conditions, proteases are packaged as precursors, and protease inhibitors are also synthetized. Nevertheless, when one of these protective mechanisms fails, acute pancreatitis occurs [4]. There are many mechanisms which could be responsible for pancreatic enzyme activation, such as pancreatic duct obstruction, that determines an accumulation of pancreatic secrete inside the pancreas, as well as acinar cell damage due to infections and/or drugs, or a defective precursor enzyme transport. Generally, the first cause of pancreatic inflammation is the presence of gallstones, followed by hypertriglyceridemia, alcohol abuse and, in a low percentage, acute pancreatitis could occur after endoscopic retrograde cholangiopancreatography or after drug treatments (i.e., azathioprine, estrogens, tetracycline, or valproic acid) [3]. The initial phase of acute pancreatitis consists of a precursor enzyme activation inside pancreatic tissue; this condition exerts a chemoattractant effect for neutrophils that trigger inflammation. If this process persists, the activated enzymes also damage other organs. Acute pancreatitis is considered a reversible condition, treated with early fluid resuscitation, analgesia and nutritional support, however, more than 50% of patients develop hyperglycemia and 5% develop diabetes. On the other hand, acute pancreatitis represents a clinical complication for type-II diabetic patients, in which the trigger factor is supposed to be the hypertriglyceridemia [7].

#### 2.2.2. Chronic Pancreatitis

When pancreatic tissue inflammation persists, acute pancreatitis progresses into the chronic form, which corresponds to a fibro-inflammatory disease characterized by irreversible changes, with the progressive destruction of the organ due to acinar atrophy, necrosis, and fibrosis [2]. The therapeutic options are very limited, and they are mainly based on pain control and on management of exocrine and endocrine insufficiency, while surgical treatments are effectively used only in selected patients [8]. Concerning etiology, oxidative stress and overexpression of inflammatory cytokines are thought to play a pivotal role for the development of chronic pancreatitis, since many authors have reported the involvement of NF-κB and Il-1β [9].

**Figure 2:**
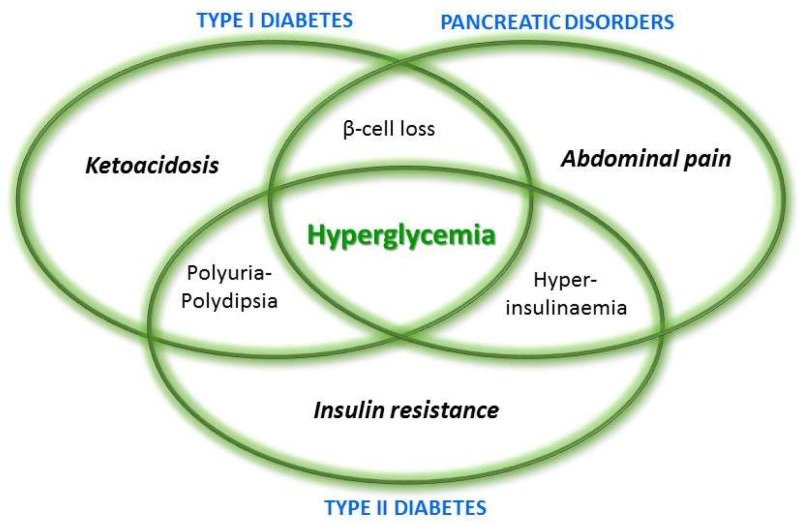
Schematic overview of shared aspects of type I–II diabetes and pancreatic disorders.

Such a progressive loss of endocrine and exocrine pancreatic tissue also leads to the onset of a peculiar diabetes form, known as type-III (c), which has an incidence higher than generally thought, since this form has been often misclassified as type-II [10,11]. Some criteria have been established in order to correctly distinguish among the different diabetes forms, which is mandatory for the right management and treatment of patients [12]. As reported above, hyperglycemia is a common aspect of all diabetes form, but it is also present in other pancreatic diseases, thus, also in acute and chronic pancreatitis, and it represents a risk factor for pancreatic cancer [13]. A schematic view of symptoms shared by type-I and type-II diabetes and pancreatic disorders is reported in Figure 2.

The etiopathogenetic aspects of the different pancreatic disorders are very disparate; nevertheless, they also present, at least, one peculiar aspect and they shared few elements in terms of metabolic signs. Certainly the common denominator of all pancreatic disorders is hyperglycemia, which is the consequence of: β-cell loss in type-I diabetes, insulin-resistance for type-II diabetes, and β-cell dysfunction due to the inflammatory process in pancreatitis. Glucose metabolism is linked to the lipid one, so the latter is influenced in cases of disorders affecting the first one. In particular, the insulin absence in type-I diabetes results in decreased lipoprotein lipase activity, which, in turn, leads to high levels of VLDL (very low density lipoprotein) and a remarkable HDL (high-density lipoprotein) increase. Generally, in these patients, when glycemia is stabilized also lipids levels return into physiological range [14]. The clinical picture of type-II diabetes includes high levels of triglycerides, which are linked to an increased hepatic synthesis of VLDL, due to an excess of substrates (glucose and fat acids) in the liver. Furthermore, an increase of FFA (free fatty acids), also correlated to type-II diabetes, is responsible for insulin resistance, which, in turn, leads to lipolysis inhibition in adipose tissue [15]. Dyslipidemia is also observed during the course of acute and chronic pancreatitis. In particular hypertriglyceridemia is itself one of the principal cause of acute pancreatitis, while, in patients affected by chronic pancreatitis, dyslipidemia is due to biliary disorder [16,17].

### 2.3. Pancreatic Cancer

Pancreatic cancer is a life-threatening disease characterized by a poor prognosis and very few effective therapeutic options. Among the different forms of pancreatic cancer, the pancreatic ductal adenocarcinoma is the most common one, representing more than 85% of cases [2]. Many authors have investigated the molecular mechanisms of pancreatic ductal adenocarcinoma, thus identifying the involvement of several oncogenic pathways, such as KRAS and Wnt oncogene activation, as well as the activation of the PI3K/Akt/mTOR pathway [2]. Moreover, the inactivation of the tumor suppressor gene PTEN has been reported [2], while the overexpression of inflammatory mediators, such as cyclooxigenase 2 (Cox2) or NF-κB, is thought to promote the transition towards neoplastic cells [9].

Concerning the possible correlation with the other pancreatic diseases, it is known that high blood glucose concentration enhances cell proliferation, at the bases of cancer development and progression, and it also increases metastatic potential. In the same way, insulin resistance and hyperinsulinaemia, occurring in Type II diabetes, as well as hyperglycemia, can promote the proliferation of pancreatic cancer [18], thus meaning that diabetes may represent a risk factor for pancreatic cancer. Due to such a bidirectional relationship between diabetes and pancreatic cancer, some authors even suggested that diabetes could represent, by itself, an early manifestation of pancreatic cancer [18].

Another aspect that must be taken into account is the correlation between pancreatitis and pancreatic cancer: it is well known that inflammation represents a risk factor for the development of tumors of different organs, including the gastrointestinal tract, the lung, and also the pancreas [19]. As reported in the literature by Munigala and colleagues, acute pancreatitis represents a risk factor, but the pathway bridging chronic pancreatitis to pancreatic cancer seems to be even more relevant [20]. Once inflammation is established, macrophages, tumor-associated macrophages, and other inflammatory cells start to release some molecules, such as cytokines, growth factors, and matrix-degrading enzymes, extrinsic signals for the generation of an environment promoting cancer formation [21]. In addition, the chronic inflammatory milieu leads to the failure of genome stability mechanisms that, in turn, enhances pancreatic tumor formation [22].

## 3. Mesenchymal Stem Cells (MSCs)

MSCs are adult stem cells firstly isolated in the 1960s [23]. Although derived from adult tissues, these cells are characterized by all the peculiar “stem cell” features, including self-renewal, plasticity, and differentiation abilities and, in addition, they also possess other singular and interesting properties, such as the capability to differentiate into multiple lineages of cells and to secrete immunomodulatory molecules [24]. Furthermore, MSCs offer several advantages: (i) They can be isolated from different tissues, such as bone marrow, adipose tissue, Wharton’s Jelly, and umbilical cords, in a relatively simple way; (ii) they are easily harvested and expanded in vitro; and (iii) they do not present ethical problems. Since devoid of a single peculiar marker, MSCs are identified and classified according to a detailed expression pattern, and to specific features (see Table 2), as stated by the International Society of Cellular Therapy guidelines [25]. Initially, the most catching feature of MSCs was the putative wide-range differentiation ability, and despite the first pioneering studies focused on such peculiarity of MSCs [26], the most intriguing property of these cells is their ability to release soluble factors. In fact, many in vitro and in vivo studies have demonstrated that MSCs are able to release a plethora of different kinds of molecules, such as pro-survival, antiapoptotic, and antiinflammatory factors, by which MSCs can interact with other cells and modify the surrounding environment [27]. Such an ability, combined with the aptitude of MSCs to migrate towards a lesion or an inflammation site, makes these cellular populations an excellent candidate for regenerative medicine purposes, in particular for pancreatic diseases. In fact, inflammation and apoptosis are key etiopathological factors of both diabetes and acute pancreatitis and, therefore, MSCs can represent the trump card of new therapeutic approaches. In addition, since a connection between T and B cells and cholesterol metabolisms [28] has been demonstrated, through their anti-inflammatory properties, MSCs can also indirectly reduce dyslipidemia, another important factor of such diseases. In addition to the evident therapeutic potential of MSCs for such diseases, there is also a less obvious, but as much promising, use for the therapy of pancreatic ductal adenocarcinoma, which will be described in the following sections.

## 4. MSCs and Diabetes

Among the studies on the MSC effect on pancreatic disorders, diabetes is certainly the most extensively investigated. As previously described, diabetes is a serious metabolic worldwide-spread disease characterized by the presence of hyperglycemia, which may be caused by an alteration of insulin production dependent on the autoimmune destruction of pancreatic β-cells (type-I diabetes), or by an altered body response to insulin (type-II diabetes) [29]. Diabetes may also occur as a result of chronic pancreatitis (type-III diabetes) [30]. In any cases, diabetic patients present a lack of insulin, with the consequent increase of blood glucose levels. The current life-saving therapy consists of exogenous insulin administration, but it is, however, unable to prevent diabetes’ long-term side effects, including nephropathy, peripheral neuropathy, and vascular alterations [29]. For this reason, several alternative therapeutic options are under examinations, such as the use of regenerative medicine and pancreatic islets transplantation. The replacement of damaged pancreatic islets with functional ones is theoretically the best options, but into the practice there are several limitations, such as early loss of grafted islets, as well as the necessity of immunosuppressive drugs and of multiple donors [29]. Concerning MSCs, they have been proposed both as a stand-alone therapy, and in combination with pancreatic islets transplantation, in order to enhance their effect and to improve their feasibility. The rationale for the use of MSCs just resides in their manifold properties: differentiation potential, immunomodulation, pro-survival effect, and anti-inflammatory factor release. Many authors have confirmed such a theory, since they demonstrated that MSC transplantation, generally after multiple injections [31,32], in diabetic animals was able to restore, or at least to increase, the insulin level, although the mechanisms remained unclear.

The immunomodulatory action of MSCs, and in particular their ability to reduce the immune system activation, could stop the production of self-antibodies against pancreatic β-cells, thus blocking their degeneration. Many authors have explored this possibility, and they have reported a change of cytokines expression pattern from a pro-inflammatory (such as TNF-α and IL-2) to an anti-inflammatory one in T cells derived from diabetic rats transplanted with MSCs [33]. In addition, MSCs resulted able both to suppress autoaggressive T cell proliferation and to enhance the production of regulatory Fox3p positive T cells [34]. Moreover, the trophic factors released by MSCs, including IL-6, IL-11, IGF-1, and VEGF, also through microvesicles [35], have been demonstrated to protect the residual endogenous pancreatic β-cells and to stimulate their regeneration, together with the proangiogenic properties of MSCs [36,37,38]. Basically, MSCs are able to produce a trophic supportive microenvironment [39]. In addition, some authors have demonstrated a spontaneous transdifferentiation of MSCs into insulin-producing cells [40,41], although this ability remains controversial, since many authors reported insulin production only after a genetic manipulation or after exposure to specific pro-differentiative factors [42,43,44].

Despite these promising data obtained with the stand-alone therapy with MSCs, the best results in achieving normoglycemia have been obtained by their transplantation in combination with pancreatic islets. There are different possible mechanisms by which MSCs could affect pancreatic islet survival and functionality. A differentiation of MSCs into insulin-releasing cells has been demonstrated in vitro after the direct contact with pancreatic islets [45]. Moreover, the release of anti-inflammatory and anti-oxidant factors can improve the engraftment and prolong the survival of transplanted pancreatic islets, as observed in different models [36,46], as well as to improve the endogenous islet regeneration already described [40,41,45,46]. Finally, a recent study demonstrated in vivo the ability of MSCs to inhibit the apoptotic pathway triggered by endoplasmic reticulum stress in transplanted pancreatic islets [47]. Taken together, these papers confirmed the promising use of MSCs for the treatment of diabetes, with the best results achieved when transplanted with pancreatic islets, and the first phase I/II clinical trials have demonstrated the safety and the feasibility of the application of MSCs (https://clinicaltrials.gov/ct2/results?cond=diabetes+and+mesenchymal+stem+cells&term=&cntry=&state=&city=&dist=), and now larger studies are necessary to validate their effectiveness for diabetes treatment [48].

## 5. MSCs and Acute and Chronic Pancreatitis

The first study which has explored the possible use of MSCs to treat acute pancreatitis dated back to 2011, with the use of the most common experimental induction model, that is, the intraductal injection of taurocholic acid (TCA) into rats [49]. In other experimental models, acute pancreatitis has been induced by the injection of molecules, such as cerulein, L-arginine, and lipopolysaccharides (LPS), as described in other papers, with comparable results [50,51]. As previously stated, the triggering event of acute pancreatitis is the intra-acinar activation of digestive enzymes, which leads to the production of inflammatory molecules, to the vacuolization and to the acinar cell death, with the consequent organ failure [49]. In all these models MSCs demonstrated to be able to reduce the pancreatitis symptoms, as well as parenchymal damage and necrosis [30]. The main protective mechanism exerted by MSCs resulted to be, as expected, their anti-inflammatory action, both through the direct release of anti-inflammatory cytokines, and through the modulation of pro-inflammatory cytokines, such as TGF-β, INF-γ, and TNF-α, which resulted in a decrease after MSC administration. In particular, Zhao and colleagues observed a migration towards pancreas of MSCs intravenously injected, followed by the decrease of IL 1-1β and of TNF-α [52]. Despite the role of MSC migration during acute pancreatitis treatment still being controversial, since it is not always observed [53], the reduction of serum levels of inflammatory cytokines after MSC injection is now assured, and it has been proposed as a regulatory mechanism of the generation of Foxp3-positive regulatory T cells and the abrogation of CD3-positive T cell proliferation [49]. This innate immunomodulatory ability of MSCs can even be strengthened by genetic manipulation: Hua and colleagues [54] demonstrated that the transfection with the angiopontin-1 gene further reduced inflammation, serum levels of amylases and lipases, as well as pancreatic injury, thus enhancing the effect of umbilical cord-derived human MSCs. Such a synergic action was probably due to the angiogenesis promotion driven by angiopontin-1 gene, which led to a vascular stabilization and to an increased survival of endothelial cells [54].

Despite the large interest on acute pancreatitis, only a few studies have explored the therapeutic potential of MSCs on chronic pancreatitis, in which the persistence of inflammation and its consequences lead to the formation of large fibrotic area, due to the transition of pancreatic stellate cells from a round shape to a fibroblast-like morphology. The presence of a fibrotic, rather than glandular parenchyma, alters and limits the pancreatic functions. In these cases, the presence of MSCs of different sources, or even of their conditioned medium, was able to reduce fibrosis and parenchymal damage, as a result of the inhibition of pancreatic stellate cells. Such an inhibition was probably achieved through the reduction of pro-inflammatory factor expression, and it was even enhanced when MSCs were previously engineered to release IκBα, an inhibitor of the pro-inflammatory molecule NF-κB [55]. In summary, MSCs resulted in being able to protect the acinar cells from the injury, as well as to reduce the inflammatory response, thus, also reducing the fibrosis.

## 6. MSCs and Pancreatic Cancer

Concerning the fight against pancreatic cancer, there are currently poor treatment options, with disabling side effects, and the search for alternative therapies is still ongoing. In this scenario, MSCs represent a manifold treatment. Since diabetes and overall pancreatitis represent risk factors for the development of pancreatic cancer, it is evident that MSCs, with their protective effect in pancreatic tissue and anti-inflammatory action, can reduce pancreatic cancer occurrence, basically simply by stopping the flow that leads to its development. In addition to this simple consideration, however, MSCs may give further advantages for the pancreatic cancer therapy, on the basis of their peculiar properties, in particular the “homing” ability (still controversial, as a matter of fact). “Homing” is the capacity of MSCs to migrate towards a lesion following the attractive force of specific chemokines released by tumor cells. By exploiting this feature, a “suicide gene therapy” with MSCs genetically engineered to produce cytotoxic molecules has been proposed. MSCs represent the ideal cells, since they have low immunogenicity and, therefore, are hardly recognized by the host immune system. Moreover, the transfection procedures, with both lentivirus or plasmids, do not seem to alter their homing ability. In this way, MSCs can be a suitable vehicle to transport directly to the tumor some inactive prodrugs or killer molecules, such as the secreting form of Trail-1, an anticancer protein able to induce apoptosis only in tumor cells, as reported by Moniri and colleagues [56]. In the same way it would be possible to engineer MSCs with antitumor genes, such as PTEN, able to downregulate the very common tumorigenic pathway PI3K/Akt/mTOR pathway, and often deleted or inactivated in many tumors [2]. In addition to the great potential deriving from genetic manipulation of MSCs, later further discussed, it should not be forgotten the intrinsic antitumor activities of these cells, which may take the dual form of soluble factor release and of direct cell-to-cell interaction. In particular, MSCs resulted in being able to spontaneously produce and release proapoptotic inhibitors of the tumorigenic Wnt signaling pathway, thus reducing cellular proliferation [57]. The same antitumor effect was achieved through a direct cell contact inhibition of the Akt pathway [58]. Therefore, MSC transfection could be used to enhance their intrinsic peculiar antitumor effects, in order to maximize their therapeutic potential for pancreatic cancer treatment, however, it should be taken into account that these kinds of procedures generally have a low efficacy, and multiple MSC injections could be required [59].

## 7. Potential Drawbacks

The first and most evident drawback for a clinical use of MSCs mainly derives on their genetic manipulation, which could theoretically change the nature of these cells, in particular when lentivirus were used. For this reason, it is pivotal to find a method allowing the removal of MSCs after the achievement of their aim, to avoid the occurrence of secondary tumors [59]. Moreover, as stated before, the transfection protocols generally have a low efficacy, thus requiring multiple injections. In addition, while it is true that MSCs possess intrinsic antitumor properties which can be enhanced by genetic manipulation, nonetheless other authors weighed in favor of a pro-tumorigenic role of MSCs, just mainly due to the pro-angiogenic and immunosuppressive abilities of these cells [2]. In the same way, it should be always kept in mind that excessive manipulation, as well as prolonged passaging, can trigger a malignant transformation of MSCs, thus imposing a series of considerations before their clinical use.

Currently, however, the most important hurdle to overcome prior to thinking about actual MSC clinical application is represented by the great variability observed among the examined papers. Different authors described different routes of administration, with MSCs from different sources, with a different delivery timing, as well as a different number of transplanted cells. However, as a matter of fact, such differences reflected in just slightly different results. It is known that the most part of MSCs intravenously injected are stopped in the lungs [60], however, Hashemian and colleagues [38]) demonstrated the similar efficacy of a different route of administration. Yang and colleagues demonstrated that the anti-inflammatory action of MSCs is time- and dose-dependent [61], however, other authors observed the same effect with different administration time protocols [49,54]. The absence of a standardized protocol is undoubtedly a serious limit for the clinical application of a MSC-based therapy, and only after the achievement of a consensus about it, together with a large scale MSC production under GMP procedures, will it be possible to use these promising cells in the clinic.

Finally, the possible autologous use is undoubtedly a very intriguing feature of MSCs. Nevertheless, it could also turn into a potential drawback, in particular in the presence of a disease. In addition to the donor’s age, which can limit the in vitro expansion of MSCs and, therefore, their amount available for transplantation [62], it is noteworthy that MSCs derived from metabolic syndrome and diabetes patients can present several problems. In fact, in such cells an increase of detrimental factors has been observed, such as oxidative stress, apoptosis, and autophagy [62], although these alterations have not been reported with MSCs from newly-diagnosed Type I diabetes [63]. All of these factors can critically affect the therapeutic application of autologous MSCs, and, therefore, the donor’s feature should be evaluated carefully.

## 8. Conclusions

**Figure 3:**
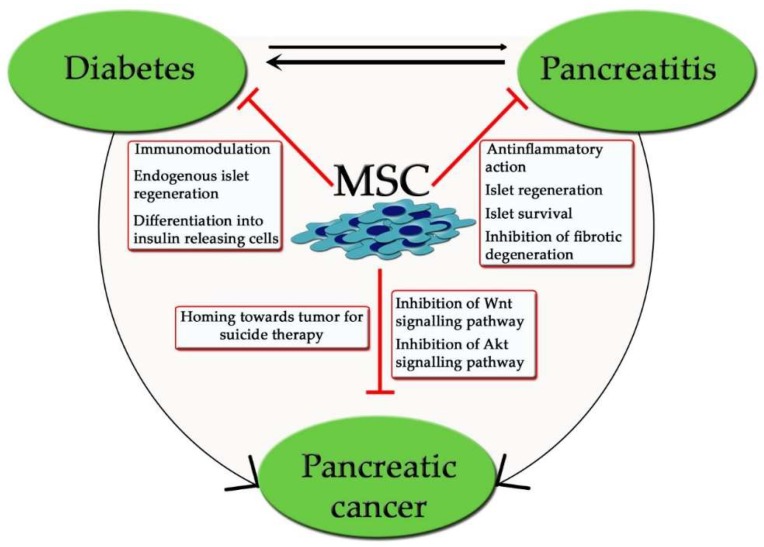
Overview of the relationships among pancreatic disorders and of MSC positive mechanisms.

Currently, MSCs represent a very promising strategy for the treatment of the three main pancreatic disorders—diabetes, pancreatitis, and pancreatic cancer—in particular pancreatic ductal adenocarcinoma. Although characterized by a different etiology and prognosis, these diseases can be strictly related. Due to their immunomodulatory and pro-survival properties, MSCs could be used to prevent disease onset or, as a cure, to protect pancreatic tissue, thus relieving disease symptoms (Figure 3). The chance to obtain engineered MSCs could further enhance their therapeutic potential, however, to move closer to their clinical application, it is necessary to identify a standard consensus protocol in order to make the most of their positive action.

## Figures and Tables

**Table 1:** Underlying mechanisms for diabetes and pancreatic disorders.

Disorder	Underlying mechanisms
**Type I Diabetes**	Immune-mediated β-cell destruction
**Type II Diabetes**	Insulin resistance and insulin deficiency
**Type III Diabetes**	a. Genetic defects β-cell function
	b. Genetic defects of insulin action
	c. Diseases of the exocrine pancreas
	d. Endocrinopathies
	e. Drug- or chemical-induced
	f. Infections
	g. Uncommon forms of immune-mediated diabetes
	h. Genetic Syndrome
**Acute Pancreatitis**	Inflammation due to pancreatic enzyme activation inside the pancreas
**Chronic Pancreatitis**	Inflammation, fibrosis and progressive destruction of exocrine and endocrine tissue
**Pancreatic Cancer**	Genetic mutations

**Table 2:** Basic criteria for MSC identification according to the ISCT guidelines.

	Positive Markers	Negative Markers
Marker Expression	CD73/5’-Nucleotidase CD90/Thy1 CD105	CD34 CD45 CD11b/CD14 CD79α HLA ClassII
Culture Type	Adhesion to plastic	
Differentiation Ability	Adipogenic differentiation, Osteogenic differentiation, Chondrogenic differentiation after specific stimulation	

Abbreviation: ISCT, International Society of Cellular Therapy.

## Abbreviations

MSCs	Mesenchymal Stem Cells
ADA	American Diabetes Association
AGEs	Advanced Glycosylation End products
PKC	Protein Kinase C
Cox2	Cyclooxigenase 2
PDK-1	Pancreatic Duodenal Homeobox-1
GLUT-4	Glucose Transporter 4
TNF-α	Tumor Necrosis Factor-α
HDL	High Density Lipoprotein
VLDL	Very Low Density Lipoprotein
FFA	Free Fatty Acids
IL	Interleukyn
IGF-1	Insulin-like Growth Factor-1
VEGF	Vascular Endothelial Growth Factor
TCA	Taurocholic Acid
LPS	Lipopolysaccharides
TGF-β	Tumor Growth Factor-β
INF-γ	Interferon-γ
Trail-1	Tumor Necrosis Factor (TNF)-related apoptosis-inducing ligand
ISCT	International Society of Cellular Therapy
